# Metformin exhibits antiproliferation activity in breast cancer via miR-483-3p/METTL3/m^6^A/p21 pathway

**DOI:** 10.1038/s41389-020-00290-y

**Published:** 2021-01-05

**Authors:** Lin Cheng, Xu Zhang, Yu-Zhou Huang, Yu-Lan Zhu, Ling-Yun Xu, Zhi Li, Xin-Yuan Dai, Liang Shi, Xu-Jie Zhou, Ji-Fu Wei, Qiang Ding

**Affiliations:** 1 Jiangsu Breast Disease Center, The First Affiliated Hospital with Nanjing Medical University, 300 Guangzhou Road, 210029 Nanjing, China; 2 Department of Breast Surgery, The Affiliated Changzhou No. 2 People’s Hospital of Nanjing Medical University, 29 Xinglong Lane, 213003 Changzhou, China; 3 Research Division of Clinical Pharmacology, The First Affiliated Hospital with Nanjing Medical University, 300 Guangzhou Road, 210029 Nanjing, China

**Keywords:** Breast cancer, TOR signalling

## Abstract

Evidence suggests that metformin might be a potential candidate for breast cancer treatment. Yet, its relevant molecular mechanisms remain to be fully investigated. We found that metformin could suppress the N6-methyladenosine (m^6^A) level in breast cancer cells significantly. The latter has an essential role in breast cancer progression and is newly considered as a therapeutic target. In this study, we measured the m^6^A level by m^6^A colorimetric analysis and dot blot assay. We then performed qRT-PCR, western blot, MeRIP, dual-luciferase reporter assay, and others to explore the m^6^A-dependent pathway associated with metformin. In vivo effect of metformin was investigated using a mouse tumorigenicity model. In addition, breast cancer and normal tissues were used to determine the role of METTL3 in breast cancer. Metformin could reduce the m^6^A level via decreasing METTL3 expression mediated by miR-483-3p in breast cancer. METTL3 is known to be able to promote breast cancer cell proliferation by regulating the p21 expression by an m^6^A-dependent manner. Metformin can take p21 as the main target to inhibit such effect. To specify, this study exhibited that metformin can inhibit breast cancer cell proliferation through the pathway miR-483-3p/METTL3/m^6^A/p21. Our findings suggest that METTL3 may be considered as a potential therapeutic target of metformin for breast cancer.

## Introduction

Over the past decade, the off-patent drug draws huge attention worldwide for repositioned as anticancer candidates. Numerous outstanding epidemiologic and clinical investigations have proved these drugs to be effective in treatment for almost all types of cancer including breast cancer^1,2^. Metformin, a widely prescribed oral glucose-lowering medication, is one of the first-line drugs for the management of type 2 diabetes mellitus (TDM2)^3,4^. As an old drug, it was firstly reported as a new treatment for breast cancer in 2005^5^. Evidence suggests that metformin can be useful in the prevention, neoadjuvant, adjuvant, extended adjuvant, and advanced disease treatment for breast cancer^6,7^. For example, a retrospective study with 2529 breast cancer patients suggested that compared with the non-metformin group, patients with TDM2 treated with metformin have a higher pathologic complete response (pCR) rate^8^. Although preclinical studies demonstrated that metformin may have different mechanisms of tumor suppression via insulin-dependent and independent pathways^9,10^, the exact molecular mechanisms for the therapeutic effect of metformin are not yet fully investigated.

One plausible speculation is that this therapeutic effect of metformin is related to N6-methyladenosine (m^6^A). M^6^A, the most prevalent internal modification on eukaryotic mRNA^11,12^, as well as “writers”, “erasers”, and “readers”, has been reported to carry essential and diverse biological functions in tumors initiation and progression for almost all types of cancer, including breast cancer^13^, gastric cancer^14^, colon cancer^15^, liver cancer^16^, and bladder cancer^17^. Recently, m^6^A regulators and related pathways have been considered as therapeutic targets for cancers^18^. Up to date, several studies identified selective and effective compounds targeting m^6^A machinery for leukemia^19–21^, glioma^22,23^, colon cancer^24^, and ovarian cancer^25^. However, few of these studies focused on breast cancer, one of the top killers of women worldwide^26^. In this paper, we investigated the relation between m^6^A and metformin and found exciting results showing that metformin could significantly suppress the level of m^6^A in breast cancer cells. To further explore this intriguing hypothesis, we performed several in vitro and in vivo experiments as following.

In the present study, we assessed levels of m^6^A and its key related genes in breast cancer cells treated with metformin. Our results showed that m^6^A level decreased after treatment of metformin. Also, methyltransferase METTL3 was the main factor involved in this aberrant m^6^A modification. Furthermore, we explored the target gene p21 of METTL3 and revealed the mechanism of modulating p21 in breast cancer.

## Materials and methods

### Breast tissue samples and cell lines

We used primary breast cancer tissues and the adjacent normal tissues from 90 patients diagnosed with breast cancer in the Affiliated Changzhou No. 2 People’s Hospital of Nanjing Medical University from 2014 to 2016. The follow-up deadline was June 2019. All patients provided written informed consent, which was conducted in accordance with the Declaration of Helsinki. The human breast cancer cells SUM1315, MCF-7, BT474, MDA-MB-231, and ZR-75-1 as well as normal mammary epithelial cells MCF-10A and HBL-100 were obtained from American Type Culture Collection (ATCC, USA) and cultured as described previously^27^.

### Transfection

Stable knockdown and overexpression of METTL3 were achieved by lentiviral-based short-hairpin RNA (shRNA) delivery. Breast cancer cells, SUM-1315, MCF-7, and BT-474 cells, were infected with METTL3 overexpression lentivirus (oeMETTL3), a negative control (oeNC), METTL3 knockdown lentivirus (shMETTL3–1, shMETTL3–2), or scrambled control (shNC), respectively. Among them, SUM-1315 and MCF-7 infected with shMETTL3–1, shMETTL3–2 and shNC were then transfected with p21 siRNA (GenePharma, China) and the nonspecific siRNA negative control. MiRNA mimics and inhibitor (GenePharma, China) were transfected using the Lipofectamine 3000 kit (Invitrogen, USA).

### Transcriptome sequencing

RNA of stable SUM-1315 METTL3 knockdown cells and scrambled control cells with three duplicates were purified and sent to a commercial company to undergo transcriptome sequencing (Allwegene, China).

### Immunohistochemistry (IHC) staining and analysis

IHC staining of the 90 breast tissue samples and tumors excised from mice with METTL3 antibody (1:200, Abcam, USA), p21 (1:200, Cell Signaling Technology, USA), and Ki-67 (1:400, Cell Signaling Technology, USA) antibodies was conducted and analyzed. The IHC scoring system was used to score the staining in breast cancer tissues as described by two pathologists^28^. METTL3 expression levels were then divided into low (0–3) and high-staining (4–9) groups.

### Quantitative real-time PCR (qRT-PCR)

Total RNA was isolated from tissues and cells using Trizol reagent (Invitrogen, USA) according to the manufacturer’s protocol. cDNA was synthesized using HiScript II (Vazyme, China). Then, qRT-PCR for mRNA and miRNA was performed on a StepOne Plus Real-Time PCR system (Applied Biosystems, USA). U6 and β-actin were used as the standard controls for miRNA and mRNA detection, respectively. All PCR primers (GenCopoeia, China) were listed in Supplementary Table S1.

### Western blot

We used the RIPA buffer to obtain the total cellular proteins and then quantified it by BCA analysis (Beyotime, China). Extracted proteins were separated by 10% SDS-PAGE and then transferred onto PVDF membranes (Millipore, USA). After the incubation with antibodies for METTL3, p21, and β-actin (1:1000, Abcam, USA) overnight, the membranes were then incubated with secondary antibody (1:1000, CST, USA). At last, target proteins were detected using a chemiluminescence system (Bio-Rad, USA).

### Cell proliferation and colony formation assay

After seeding cells with/without metformin treatment of different time durations, we measured cell growth by CCK-8 assay kit (Dojindo, Japan). The absorbance was measured at 450 nm using a microplate reader (Tecan, Switzerland). For colony formation assay, 500 cells per well were seeded into 6-well plates and maintained for 2 weeks. Finally, the cells were stained with Giemsa (Sigma, USA) for 30 min, the colonies containing 50 or more cells were counted.

### Cell cycle assay

1 × 10^6^ cells treated with/without metformin were collected, washed with PBS, and fixed with 75% ethanol at −20 °C overnight. The cells were then stained with propidium iodide (BD Biosciences, USA) and assessed by flow cytometry (Becton Dickinson, USA).

### RNA m^6^A quantification

For m^6^A dot blot assay, the poly (A)^+^ RNA from cells was firstly spotted onto a nylon membrane (GE Healthcare, USA), then the membranes were UV cross-linked (254 nm), blocked, incubated with m^6^A antibody (1:1000, Abcam, USA) at 4 °C for 24 h. Subsequently, the membranes were incubated with goat anti-mouse IgG (1:3000, Proteintech, USA). Finally, the membrane could be visualized using the chemiluminescence system (Bio-Rad, USA). Moreover, EpiQuik m^6^A RNA Methylation Quantification Kit (Epigentek, USA) was used to detect the m^6^A level in total RNAs from tissues.

### Dual-luciferase reporter assay

We transfected cells with plasmids containing wild or mutant fragments from METTL3 or p21 using Lipofectamine 3000 according to the protocol. Using a dual-luciferase reporter assay system (Promega, USA), the firefly and renilla luciferase activities were measured consecutively after transfection for 24 h.

### M^6^A RNA immunoprecipitation assay (MeRIP)

Total RNA of METTL3 knockdown and negative control SUM-1315 cells was isolated. The poly (A)^+^ RNA was further enriched using the mRNA Purification Kit (Invitrogen, USA) and treated with DNase I (Sigma, USA). Then, it was fragmented by sonication for 10 s on an ice-water mixture. Next, DNA-free fragmented RNA was incubated with m^6^A antibody for immunoprecipitation using Magna methylated RNA immune-precipitation (MeRIP) m^6^A Kit. Finally, the extracted RNA was subjected to qRT-PCR using primers for p21 and normalizing to Input.

### RNA stability assay

METTL3 knockdown and overexpression cells, as well as their control cells, were cultured in 6-well plates. Then actinomycin D (Act D, 5 μg/ml) was added to the cells at 0, 2, 4, and 8 h before collection. Total RNA was isolated and qRT-PCR was performed to measure the relative level of p21 as mentioned previously^29^.

### Xenografts in mice

About 1 × 10^7^ stable METTL3 overexpression and negative control SUM-1315 cells were injected subcutaneously into the axilla of the female BALB/C nude mice (4–6 weeks old, 18–20 g, 10 mice/group). One week after injection, the two groups, METTL3 OE and NC, were then randomly allocated into the control group and experimental group (5 mice/group), which were treated with PBS or metformin (250 mg/kg/dose, respectively). PBS or metformin was administered every 2 days via intraperitoneal injection.

Tumor volume and weight were measured every week. Tumor volume was calculated with the formula: volume = (length × width^2^)/2. Four weeks after injection with/without metformin, we sacrificed the mice and excised the tumors, then the tumors were weighed, portions of which were fixed in paraformaldehyde and frozen in liquid nitrogen for further analysis. The in vivo experiments were performed in accordance with the guide for the use of laboratory animals.

### Statistical analysis

Data were analyzed using SPSS version 20.0 and presented as means ± standard deviation (SD). Student’s *t*-test and one-way ANOVA were used to analyze the differences between the two groups while *χ*^2^ test was used to assess the correlation between METTL3 and the clinicopathological parameters. *P* < 0.05 was used to indicate statistical significance.

## Results

### Metformin reduced the m^6^A abundance via decreasing METTL3 expression

**Fig. 1:**
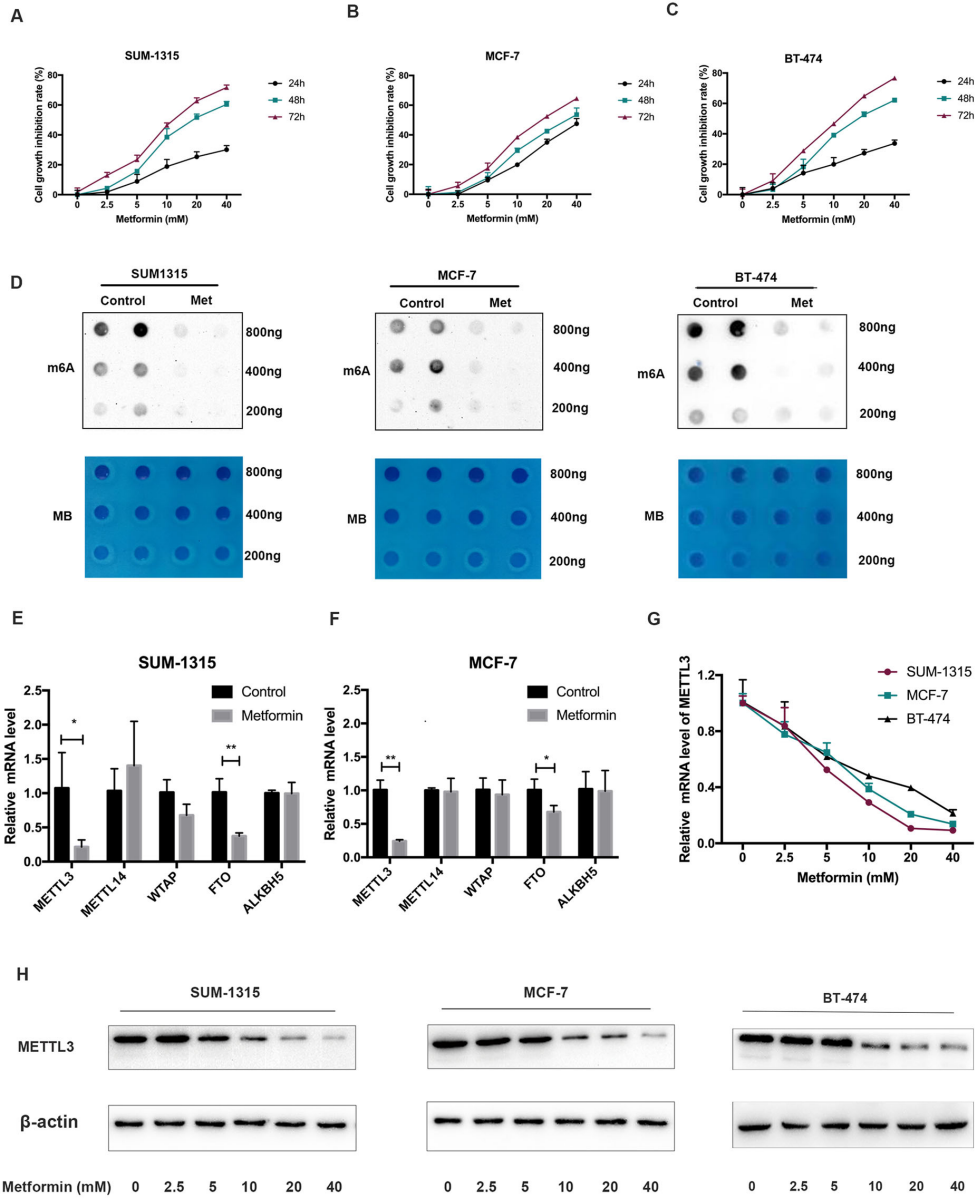
Metformin reduced the m^6^A level via decreasing METTL3 expression. **A**–**C** Metformin inhibited the growth of SUM-1315, MCF-7, and BT-474 cells in both dose-dependent and time-dependent manners. **D** Dot-blot assay showed that metformin decreased the m^6^A level in SUM-1315, MCF-7, and BT-474 cells. MB, methylene blue staining (loading control). **E**, **F** RNA expression changes of the 5 key m^6^A methyltransferases and demethylases including METTL3, METTL14, WTAP, FTO, and ALKBH5 after the treatment of metformin in SUM-1315 and MCF-7 cells. **G**, **H** Metformin decreased the mRNA and protein expression of METTL3 in a dose-dependent manner. The relative RNA level was calculated by the 2^−ΔΔCt^ method and normalized based on β-actin. The average mRNA level of genes mentioned above in the metformin untreated group was set as 1. Data represented the mean ± SD, *p < 0.05, **p < 0.01 vs metformin-untreated group.

It was reported previously that metformin could inhibit the proliferation of breast cancer cells^30^. We repeated the same experiments and found that metformin indeed inhibited the growth of SUM-1315, MCF-7, and BT-474 cells in both dose-dependent and time-dependent manners (Fig. 1A–C). These breast cancer lines could represent different subtypes of breast cancer. Additionally, we found that metformin treatment significantly decreased m^6^A level in SUM-1315, MCF-7, and BT-474 cells as detected by m^6^A dot blot assay (Fig. 1D). The m^6^A modifications are primarily affected by m^6^A methyltransferase including METTL3, METTL14, WTAP, and demethylase FTO and ALKBH5^31^. We then hypothesized that the abnormal m^6^A modification induced by metformin was caused by the dysregulation of the key m^6^A methyltransferases and demethylases. Expression changes of m^6^A related genes including METTL3, FTO, and WTAP in MCF-7 cells were observed, when treated with metformin (from GSE69845 database). We further discovered that metformin could decrease both METTL3 and FTO mRNA expression in SUM-1315 and MCF-7 cells (Fig. 1E, F). METTL3 was then selected as the candidate molecule for aberrant m^6^A modification caused by metformin since it exhibits a positive correlation with m^6^A level. Indeed, metformin could decrease the mRNA and protein expression of METTL3 in SUM-1315, MCF-7, and BT-474 cells in a dose-dependent manner after its treatment for 48 h (Fig. 1G, H).

### METTL3 was upregulated in breast cancer and correlated with worse prognosis of breast cancer patients

**Fig. 2:**
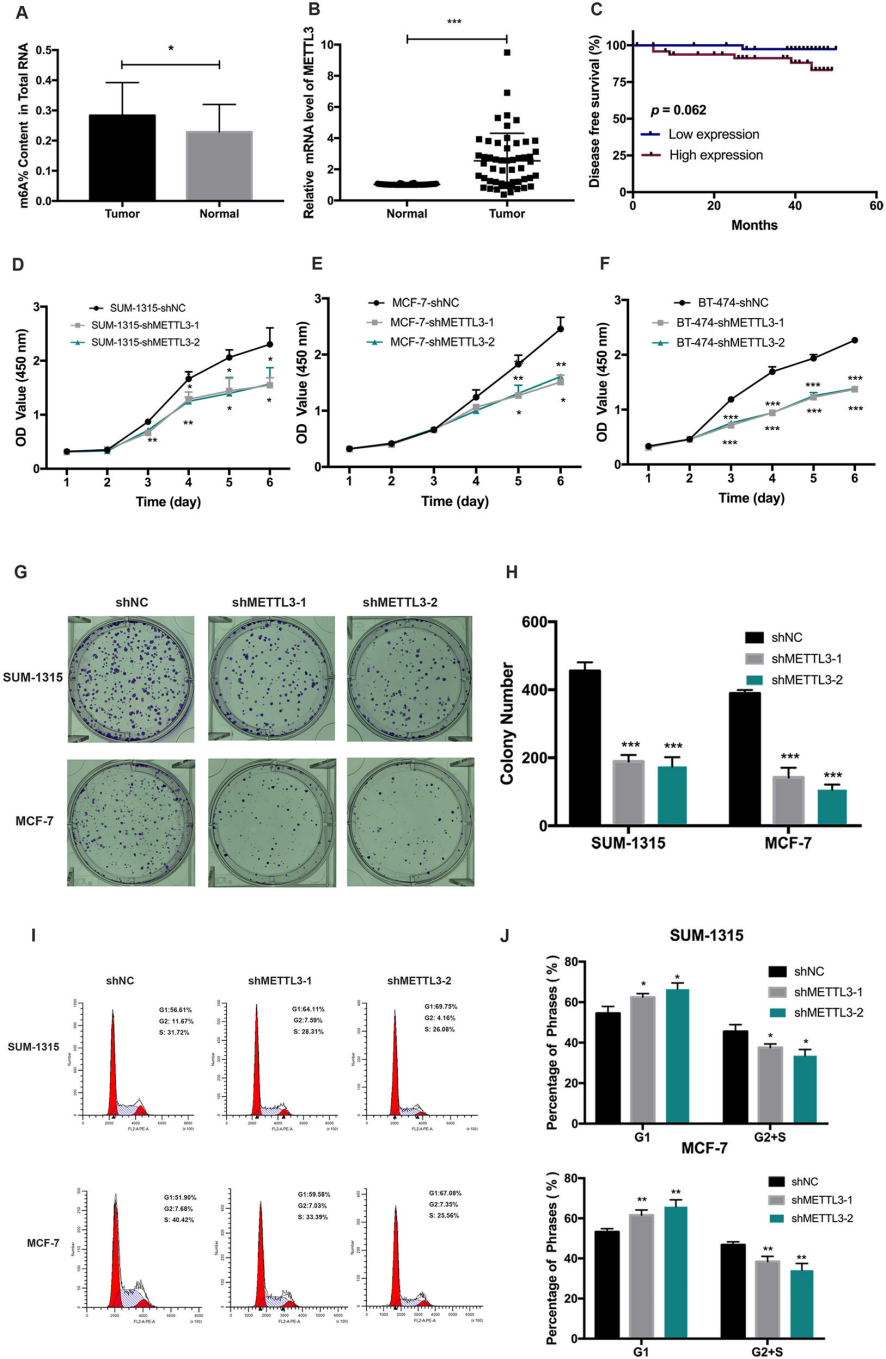
METTL3 promoted breast cancer cell proliferation. **A** M^6^A level was higher in breast cancer tissues (tumor) than adjacent normal tissues (normal) (*n* = 32). **B** The mRNA expression of METTL3 was higher in breast cancer tissues (tumor) than adjacent normal tissues (normal) (*n* = 90), ***p < 0.001. The relative RNA level was calculated by the 2^−ΔΔCt^ method and normalized based on β-actin. The average mRNA level of adjacent normal tissues was set as 1. **C** Kaplan–Meier survival curves of disease-free survival (DFS) in 90 breast cancer patients based on METTL3 expression showed that higher expression of METTL3 tended to be correlated with shorter DFS of breast cancer patients. The log-rank test was used to compare the difference between the two groups (*p* = 0.062). **D**–**F** Knockdown of METTL3 significantly decreased the growth of SUM-1315, MCF-7, and BT-474 cells by CCK-8 assay. *p < 0.05, **p < 0.01, ***p < 0.01 vs. scrambled control group (shNC). **G**, **H** Knockdown of METTL3 decreased the colony formation efficiency in SUM-1315 and MCF-7 cells. ***p < 0.001 vs. scrambled control group (shNC). **I**, **J** Cell cycle analyzed by flow cytometry. Histogram showed that METTL3 knockdown arrested at the G1 phase in SUM-1315 and MCF-7 cells. *p < 0.05, **p < 0.01 vs. scrambled control group (shNC). Data represented the mean ± SD.

We conducted an m^6^A colorimetric analysis and observed a significant increase of global m^6^A level in 32 fresh human breast cancer tissues compared to that in adjacent normal tissues (Fig. 2A). METTL3 mRNA expression in breast cancer tissues was significantly higher than that in adjacent normal tissues (Fig. 2B). Moreover, METTL3 was upregulated in breast cancer cells, compared with that in the normal breast epithelial cell lines MCF-10A and HBL-100 in both mRNA and protein levels (Supplementary Fig. S1A, B). MDA-MB-231 cells expressed a very low amount of METTL3 (Supplementary Fig. S1A, B) and the cell growth inhibition was weaker than other breast cancer cell lines significantly (Supplementary Fig. S1C). IHC analysis showed that the expression of METTL3 in breast cancer tissues was positively related to tumor size and TNM grade (Supplementary Table S2). Furthermore, using Kaplan–Meier analysis, we also found that breast cancer patients with higher expression of METTL3 had a tendency of worse prognosis and shorter disease-free survival, compared with those with lower expression of METTL3. But it had no statistical significance (*p* = 0.062) (Fig. 2C).

### METTL3 promoted the proliferation of human breast cancer cells

SUM-1315, MCF-7, and BT-474 cells were stably transfected with knockdown lentivirus and negative control lentivirus, which were then confirmed by qRT-PCR and western blot (Supplementary Fig. S1D, E). The CCK-8 and colony formation assay displayed that METTL3 knockdown led to significantly decreased cell proliferation (Fig. 2D–F) and colony formation efficiency (Fig. 2G, H). Furthermore, the flow cytometry analysis showed that the percentage of cells in the G1 phase increased while the percentage of that in the G2 and S phases decreased in METTL3 knockdown cells (Fig. 2I, J).

SUM-1315, MCF-7, and BT-474 cells were stably transfected with overexpression lentivirus, and negative control lentivirus, which were then confirmed by qRT-PCR and western blot (Supplementary Fig. S1F, G). Overexpression of METTL3 significantly promoted cell proliferation (Supplementary Fig. S2A–C) and colony formation efficiency (Supplementary Fig. S2D, E). Furthermore, the flow cytometry analysis showed that the percentage of cells in the G1 phase decreased while the percentage of cells in the G2 and S phases increased in METTL3 overexpression cells (Supplementary Fig. S2F, G).

### Metformin inhibited breast cancer cell proliferation by downregulating METTL3

**Fig. 3:**
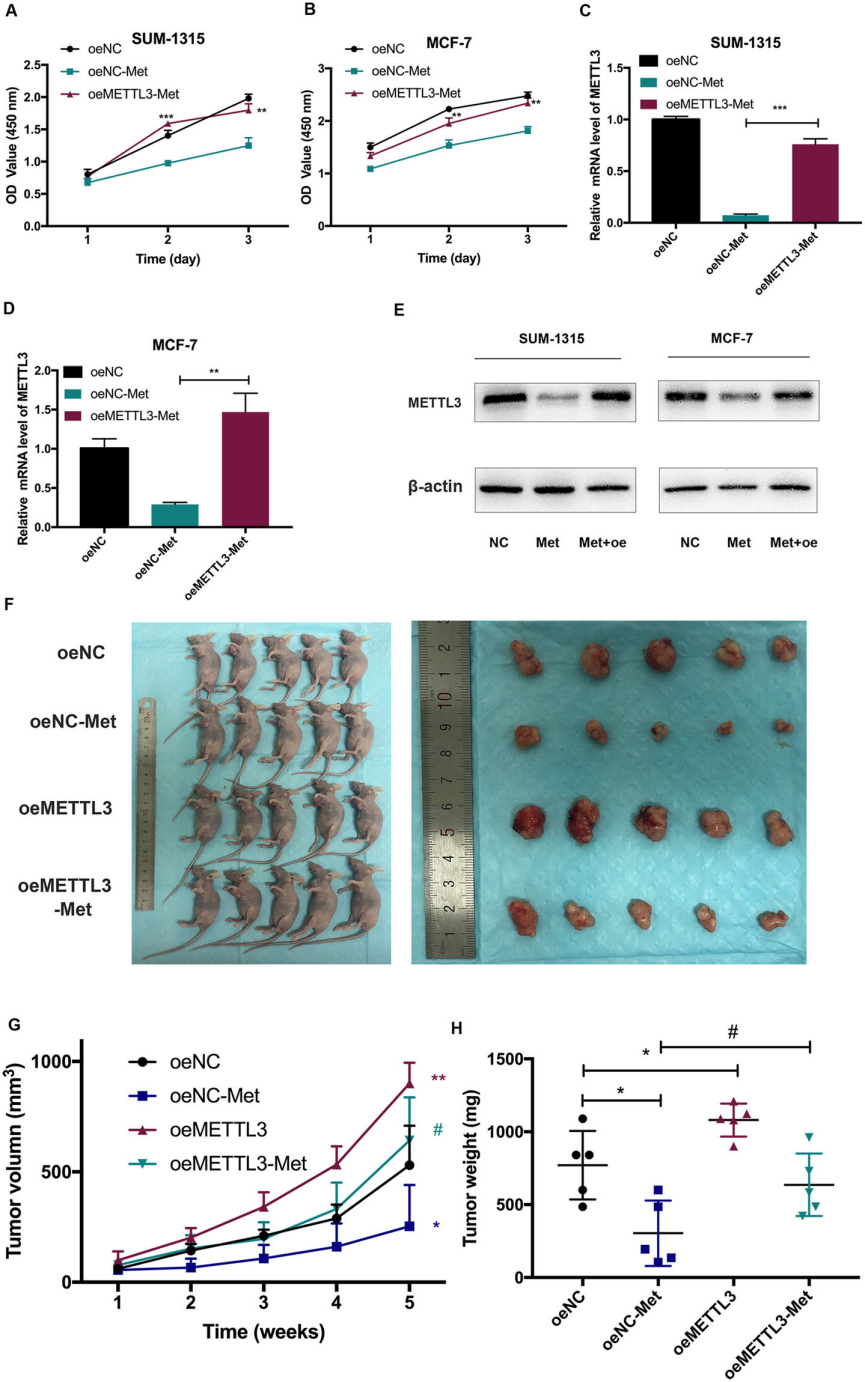
Metformin inhibited breast cancer cell proliferation by downregulating METTL3. **A**, **B** Overexpression of METTL3 attenuated the cell growth inhibitory effect of metformin in SUM-1315 and MCF-7 cells by CCK-8 assay. **C**–**E** Overexpression of METTL3 attenuated the expression inhibition of METTL3 induced by metformin both in mRNA and protein level in SUM-1315 and MCF-7 cells. **F**–**H** Overexpression of METTL3 attenuated the inhibitory effects of metformin-inhibited tumor growth in vivo. Tumor weight and the tumor growth curve were measured in the four groups. Representative photographs (**F**) and quantification (**G**, **H**) were shown. oeNC, negative control; oeNC-Met, oeNC-Met, negative control group treated with metformin; oeMETTL3, METTL3 overexpression; oeMETTL3-Met, METTL3 overexpression group treated with metformin. Data represented the mean ± SD, *p < 0.05, **p < 0.01, ***p < 0.001 vs. oeNC grou*p*, ^#^*p* < 0.05 vs. oeNC-Met group.

Overexpression of METTL3 largely attenuated the growth inhibitory effect induced by metformin (Fig. 3A, B). Accordingly, overexpression of METTL3 largely attenuated the inhibition of METTL3 induced by metformin, both in mRNA and protein level (Fig. 3C–E).

In vivo, the tumors from METTL3 overexpression cells grew more rapidly and the tumor weights were heavier, compared with the control cells (Fig. 3F–H). We also performed the rescue experiment in vivo. Metformin (250 mg/kg) significantly inhibited tumor growth after 4 weeks of intraperitoneal injection, exhibiting lower tumor weights and smaller tumor sizes. Overexpression of METTL3 could significantly reduce the inhibitory effect of metformin in tumor growth (Fig. 3F–H).

### Metformin upregulated the expression p21 by reducing METTL3-associated m^6^A modification

**Fig. 4:**
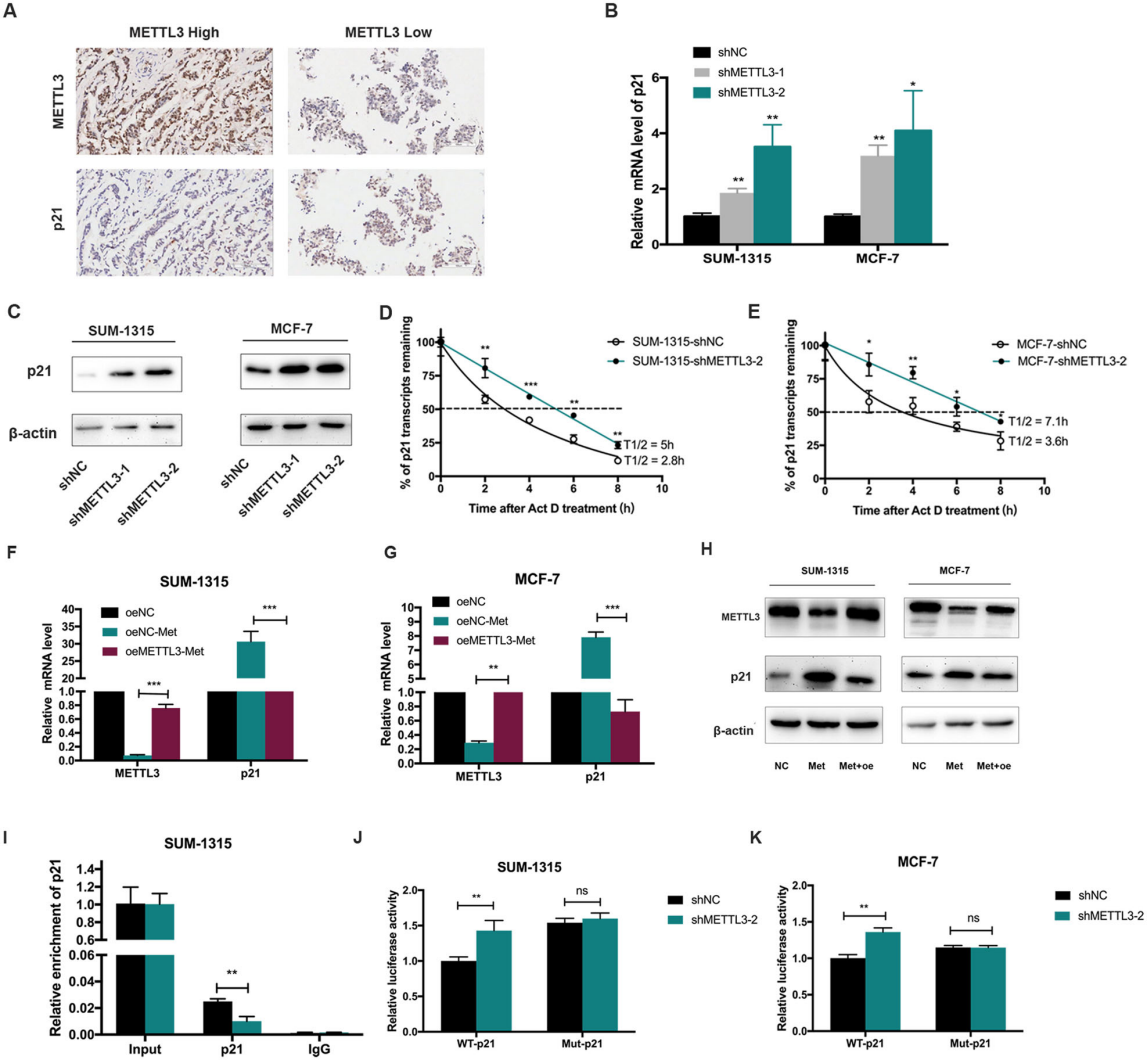
Metformin upregulated the expression of p21 by reducing METTL3-associated m^6^A modification. **A** The breast cancer tissue with the high staining of METTL3 expressed a low level of p21 while the tissue with the low staining of METTL3 expressed a high level of p21. **B**, **C** The mRNA and protein expression of p21 was upregulated after METTL3 knockdown in SUM-1315 and MCF-7 cells. The relative RNA level was calculated by the 2^−ΔΔCt^ method and normalized based on β-actin. The average mRNA level of p21 in shNC group was set as 1. **D**, **E** Knockdown of METTL3 prolonged the half-life of p21 transcript in SUM-1315 and MCF-7 cells. **F**–**H** Overexpression of METTL3 attenuated the regulation of METTL3 and p21 by metformin both in mRNA and protein level in SUM-1315 and MCF-7 cells. The relative RNA level was calculated by the 2^−^^ΔΔCt^ method and normalized based on β-actin. The average mRNA level of METTL3 and p21 in oeNC group was set as 1. **p < 0.01, ***p < 0.001 vs. oeNC-Met group. **I** MeRIP followed by qRT-PCR showed that METTL3 knockdown could decrease the amount of p21 mRNA modified by m^6^A. The relative enrichment level of p21was calculated by the 2^−^^ΔCt^ method and normalized based on Input. **p < 0.01 vs. shNC grou*p*. **J**, **K** After METTL3 knockdown, luciferase activity of wild-type 3′-UTR of p21 was increased in SUM-1315 and MCF-7 cells while mutant abolished this induction. **p < 0.01 vs. shNC group, ns means not significant. shNC, scramble control; shMETTL3 1/2, METTL3 knockdown; oeNC, negative control; oeNC-Met, oeNC-Met negative control group treated with metformin; oeMETTL3-Met, METTL3 overexpression group treated with metformin; WT-p21, wild-type 3′-UTR of p21, Mut-p21, mutant-type 3′-UTR of p21. Data represented the mean ± SD.

Transcriptome sequencing analysis identified p21 as a downstream target of METTL3 (Supplementary Results, Supplementary Tables S3, S4, and Supplementary Fig. S3A–F). IHC staining showed that the expression of p21 was negatively correlated with METTL3 (Fig. 4A and Supplementary Table S2). To verify p21 as a downstream target of METTL3, we tested the mRNA and protein expression level of p21 in breast cancer cells. In agreement with previous mRNA sequencing data, p21 was significantly upregulated in stable METTL3 knockdown SUM-1315 and MCF-7 cells (Fig. 4B, C) and downregulated in METTL3 overexpression cells (Supplementary Fig. S4A, B).

The half-life of p21 mRNA increased after METTL3 knockdown (Fig. 4D, E) and decreased after METTL3 overexpression (Supplementary Fig. S4C, D), suggesting that METTL3 could decrease p21 mRNA stability in breast cancer cells. Overexpression of METTL3 could attenuate the regulation of METTL3 and p21 caused by metformin in both mRNA and protein levels (Fig. 4F–H). Metformin could decrease the expression of p21 in a dose-dependent manner in MCF-7, SUM-1315, and BT-474 cells (Supplementary Fig. S4E). Moreover, using anti-m^6^A antibody, we found that METTL3 knockdown could decrease the amount of m^6^A-modified p21 mRNA (Fig. 4I). To determine whether p21 was a direct target of METTL3, we performed the luciferase reporter assay with p21 3′-UTR containing wild-type or mutant m^6^A sites (GGACT deletion mutation). A significant induction in the luciferase activity was observed in the wild-type 3′-UTR of p21 in METTL3 knockdown cells compared with control cells, while the mutant one almost abolished this induction, indicating that the expression of p21 was under the control of METTL3 associated m^6^A modification (Fig. 4J, K).

### METTL3 promoted breast cancer cell proliferation by mediating p21 expression

**Fig. 5:**
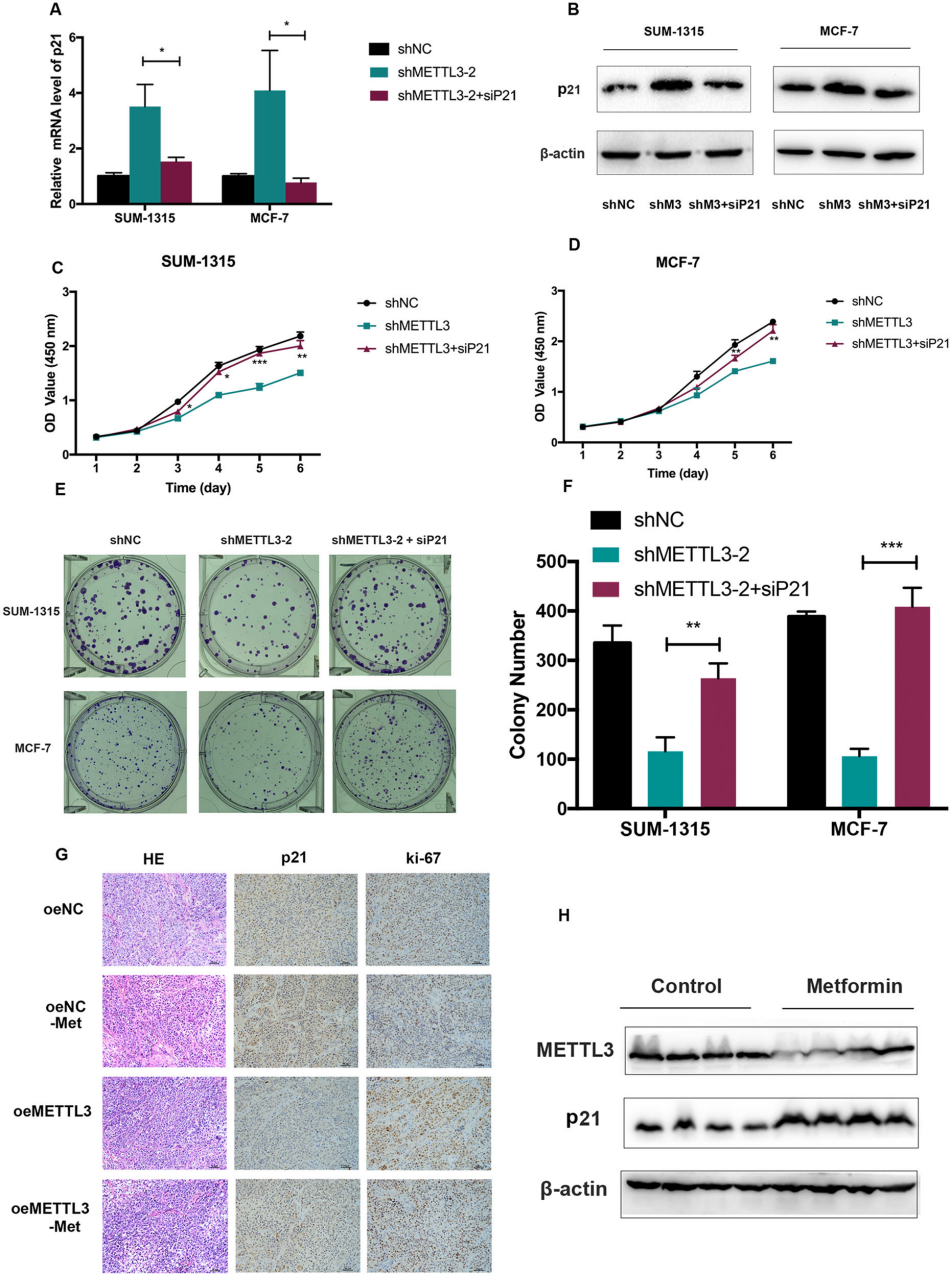
METTL3 promoted breast cancer cell proliferation by mediating p21 expression. **A**, **B** Silencing of p21 inhibited the increasing of p21 induced by METTL3 knockdown in both RNA and protein level in SUM-1315 and MCF-7 cells. The relative RNA level was calculated by the 2^−^^ΔΔCt^ method and normalized based on β-actin. The average mRNA level of p21 in the shNC group was set as 1. **C**–**F** Suppression of p21 alleviated the inhibitory effects on cell proliferation and colony formation mediated by METTL3. **G** IHC of subcutaneous xenograft tumors showed that a significant increase in the positive rate of Ki-67 while a significant decrease in the positive rate of p21 in the METTL3 overexpression group. **H** The protein level of METTL3 in the tumors of mice treated with metformin decreased than those with PBS, while the protein level of p21 increased in the metformin group. shNC, scramble control; shM3/shMETTL3-2, METTL3 knockdown; shM3 + siP21, METTL3 knockdown cells transfected with p21 siRNA; oeNC, negative control; oeNC-Met, oeNC-Met negative control group treated with metformin; oeMETTL3, METTL3 overexpression; oeMETTL3-Met, METTL3 overexpression group treated with metformin. Data represented the mean ± SD, *p < 0.05, **p < 0.01 vs. shMETTL3-2 group.

We synthesized a distinct small interfering RNA (siRNA) targeting p21 (siP21) in METTL3 stable knockdown breast cancer cells to silence p21 in SUM-1315 and MCF-7. Silencing p21 could inhibit the increased expression of p21 induced by METTL3 knockdown in both mRNA and protein levels (Fig. 5A, B). We found that reduction of p21 could alleviate the cell proliferation inhibitory effect induced by METTL3 knockdown (Fig. 5C, D). Consistently, a similar phenomenon was confirmed by the colony formation assay in SUM-1315 and MCF-7 cells (Fig. 5E, F).

Furthermore, we detected the mRNA expression of METTL3 and p21 in tissues from mice xenograft model. The mRNA level of METTL3 in the tumors from METTL3 overexpression cells significantly increased than those from control cells (Supplementary Fig. S4F), while the mRNA level of p21 in the tumors from METTL3 overexpression cells significantly decreased than those from control cells (Supplementary Fig. S4G). IHC of subcutaneous xenograft tumors showed that a significant increase in the positive rate of Ki-67 and a significant decrease in the expression of p21 in the METTL3 overexpression group (Fig. 5G). In addition, the protein level of METTL3 in the tumors of mice treated with metformin decreased than those with PBS, while the protein level of p21 in the tumors of mice treated with metformin increased than those with PBS (Fig. 5H). Taken together, we found that METTL3 promoted breast cancer cell proliferation by mediating p21 expression.

### Metformin decreased the expression of METTL3 in breast cancer cells by targeting miR-483-3p

**Fig. 6:**
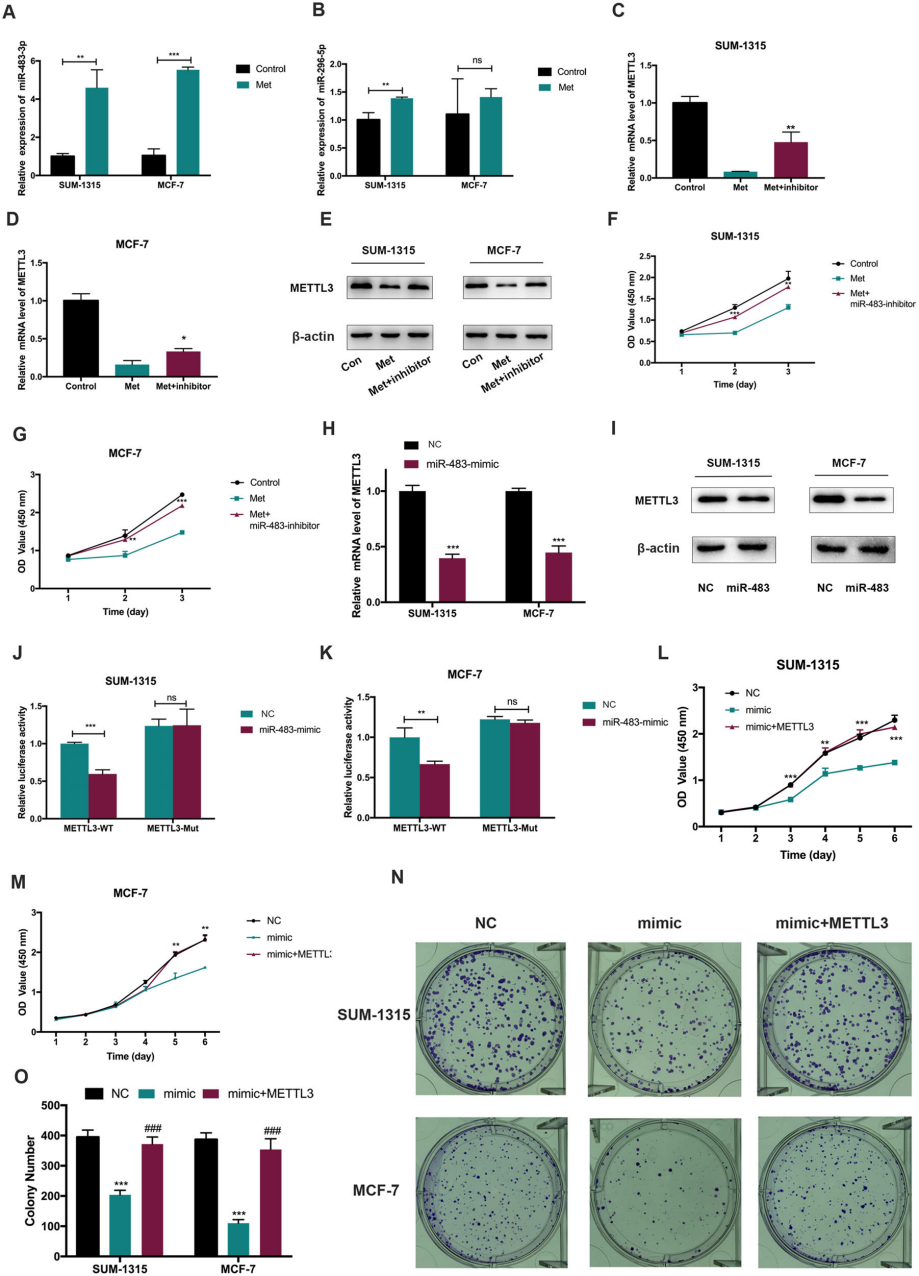
Metformin inhibited the expression of METTL3 in SUM-1315 and MCF-7 cells by targeting miR-483-3p. **A**, **B** Metformin upregulated miR-483-3p markedly in both SUM-1315 and MCF-7 cells and miR-596-5p moderately in only SUM-1315 cells. The relative expression of miRNA was calculated by the 2^−ΔΔCt^ method and normalized based on U6. The average miRNA level in the metformin untreated group was set as 1. **p < 0.01, ***p < 0.001 vs. metformin untreated group. **C**–**G** Inhibitor of miR-483-3p could counteract metformin-induced METTL3 downregulation and cell growth inhibition in SUM-1315 and MCF-7 cells. The relative expression of miRNA was calculated by the 2^−^^ΔΔCt^ method and normalized based on U6. The average miRNA level in the metformin untreated group was set as 1. *p < 0.05, **p < 0.01, ***p < 0.001 vs. metformin-treated group. **H**, **I** MiR-483-3p mimics decreased the METTL3 expression both in mRNA and protein levels. The relative RNA level was calculated by the 2^−ΔΔCt^ method and normalized based on β-actin. The average mRNA level of p21 in the NC group was set as 1. ***p < 0.001 vs. NC group. **J**, **K** MiR-483-3p mimic only inhibited the luciferase activity of the METTL3 wild-type reporter (not Mutant-type) in SUM-1315 and MCF-7 cells. **p < 0.01, ***p < 0.001 vs. nc-mimic group. **L**–**O** CCK-8 and colony formation assays showed that miR-483-3p mimic could inhibit the growth of SUM-1315 and MCF-7 cells and overexpression of METTL3 rescued the inhibitory effect of miR-483-3p. ***p < 0.001 vs. NC group, ^#^*p* < 0.05, ^##^*p* < 0.01, ^###^*p* < 0.001 vs. mimic group. Met, metformin; Con, control; ns, not significant. Data represented the mean ± SD.

Bioinformatics analysis (http://starbase.sysu.edu.cn) was used to identify potential miRNAs involved in METTL3 regulation and found 49 miRNAs potentially interact with the 3′-UTR of METTL3 mRNA. We further analyzed the miRNA expressions from MCF-7 treated with metformin (GSE37038) and selected 6 miRNAs. Considering the definite “3p” or “5p” of miRNA, we chose two of them (miR-483-3p and miR-596-5p) as candidates. We further found that metformin upregulated miR-483-3p markedly in SUM-1315 and MCF-7 cells and miR-596 moderately in only SUM-1315 cells (Fig. 6A, B). We then focused on miR-483-3p for the rest of the research and found that miR-483-3p was downregulated in breast cancer cells, compared with that in the normal breast epithelial cell lines MCF-10A and HBL-100 (Supplementary Fig. S4H).

To determine the role of miR-483-3p in the downregulation of METTL3 in breast cancer cells, SUM-1315 and MCF-7 cells transfected with/without miR-483-3p inhibitor were treated with/without metformin, and the mRNA and protein expression of METTL3 were determined. It showed that metformin led to lower METTL3 expression and inhibitor of miR-483-3p could counteract METTL3 downregulation induced by metformin (Fig. 6C–E). Moreover, an inhibitor of miR-483-3p could counteract metformin-induced cell growth inhibition (Fig. 6F, G). Transfection of breast cancer cells with miR-483-3p mimics led to a significant reduction of the METTL3 expression both at the mRNA and protein levels (Fig. 6H, I). In addition, we constructed luciferase reporters harboring the 3′-UTR of METTL3 with either wildtype (Wt) or mutant (Mut) miR-483-3p binding site. The SUM-1315 and MCF-7 cells were transiently co-transfected with miR-483-3p mimic and the luciferase activities of reporter plasmid were measured. It showed that miR-483-3p mimic only inhibited the luciferase activity of the Wt reporter (not Mut), suggesting that METTL3 was targeted by miR-483-3p in breast cancer cells (Fig. 6J, K). Furthermore, CCK-8 and colony formation assay showed that miR-483-3p mimic could inhibit the growth of breast cancer cells, and overexpression of METTL3 rescued the growth inhibitory effect of miR-483-3p among breast cancer cell lines (Fig. 6L–O). These data demonstrated metformin decreased the expression of METTL3 in breast cancer cells by targeting miR-483-3p.

## Discussion

Epidemiological studies have found an association between metformin and a beneficial effect on cancer prevention and treatment, which lead to an explosion of interest in the potential use of metformin as an anticancer drug^10^. For the molecular mechanisms in breast cancer, metformin activates AMP-activated protein kinase (AMPK) and leads to the inhibition of mTOR signaling and protein synthesis, which is responsible for the cancer cell proliferation^32^. It can also sensitize the tissues to insulin, reduce hepatic gluconeogenesis, and lower the circulating insulin level, indirectly having an anticancer role^33^. The anti-breast cancer activity of this drug follows indirect and direct pathways as mentioned above, a few of which are closely related to RNA modification levels^34^. In the present study, we found that metformin could reduce the m^6^A level via decreasing METTL3 expression mediated by miR-483-3p in breast cancer. Moreover, p21 is the main target of METTL3 in the breast cancer proliferation inhibitory effect of metformin. METTL3 could promote breast cancer cell proliferation by regulating the p21 expression by an m^6^A-dependent manner. This study exhibited the pathway of miR-483-3p/METTL3/m^6^A/p21 involved in the anti-breast cancer activity of metformin (Supplementary Fig. S5).

Two independent previous studies showed that METTL3 had an oncogenic role in breast cancer through the diverse downstream targets such as tumor suppressor let-7g^13^ and bcl-2^35^. However, some bioinformatics analysis showed that METTL3 was decreased in the breast cancer tissues and associated with better survival^36^. The dual role of METTL3 in other cancers was also reported. For instance, METTL3 promoted the growth of human liver cancer cells^16^ and gastric cancer cells^37^ but also acted as a tumor suppressor in renal cell carcinoma^38^. In the present study, we found that METTL3 was significantly upregulated in breast cancer cell lines and tissues. Knockdown of METTL3 inhibited the proliferation of breast cancer cells, whereas overexpression of METTL3 promoted the proliferation of these cells. Moreover, in vivo experiments showed that overexpression of METTL3 in SUM-1315 cells formed larger tumors in nude mice. In addition, higher expression of expression was associated with the higher TNM grade and larger tumor size as well as worse prognosis in breast cancer patients. These results were in line with the above-mentioned reports suggesting that METTL3 may act as an oncogene in breast cancer. It is important to note the limitations of the current study. To specify, the sample sizes were relatively small and the findings should be further tested with larger sample sizes.

In this study, we found that metformin could reduce the m^6^A level via decreasing METTL3 expression in breast cancer. M^6^A modification and its related proteins have the potential as drug targets in the treatment of various cancers^39^. However, regulators or inhibitors of m^6^A modifications are seldom reported till now, most of which are demethylase FTO inhibitors^22,40^. Among them, as the only FTO inhibitor that has been tested through systemic administration in mice, R-2HG inhibited FTO activity and increased global m^6^A modification, which in turn decreased c-Myc and CEBPA m^6^A modification, leading to leukemia cell growth inhibition, cell-cycle arrest, and apoptosis^20^. METTL14 had an oncogenic role by regulating the MYB/ MYC axis through m^6^A modification and SPI1 could directly inhibit the expression of METTL14 in malignant hematopoietic cells^41^. Regulators of other m^6^A methyltransferases and their cofactors are currently scarce^42^. METTL3 might be a therapeutic target, regarding its oncogenic role in breast cancer. Our study is one of the first studies to report that metformin showed antiproliferation effect on breast cancer as a novel METTL3 inhibitor. Blandino et al. revealed a change of the overall microRNA levels of breast cancer cells treated with metformin. MicroRNA modulation could be part of the mechanism of metformin for its anti-breast cancer activity^43,44^. Through bioinformatic analysis and verified experiments, we focused on miR-483-3p as a possible target microRNA of metformin and an upstream microRNA of METTL3, suggesting that metformin inhibited the proliferation of breast cancer cells by the miR-483-3p/METTL3 pathway. Tumor suppressor function of miR-483-3p by targeting different genes on breast cancer has been reported recently^45,46^. Metformin inhibited the expression of METTL3 and growth of breast cancer cells and the effect could be rescued by a miR-483-3p inhibitor, which was consistent with those previous findings discussed above. Taken together, metformin could reduce the m^6^A level via decreasing METTL3 expression mediated by miR-483-3p in breast cancer. MDA-MB-231 cells expressed a very low amount of METTL3 and cell growth inhibition by metformin was weaker than other breast cancer cell lines, further confirming the specificity of miR-483-3p/METTL3 axis as a target for metformin in breast cancer.

By analyzing transcriptome sequencings for MCF-7 cells treated with metformin and METTL3 knockdown SUM-1315 cells, we found that p21 might be the main target of METTL3 in breast cancer proliferation inhibitory effect of metformin. Knockdown of METTL3 could promote the p21 expression in both mRNA level and protein level and overexpression of METTL3 could attenuate the regulation of METTL3 and p21 by metformin both in mRNA and protein level. We then verified the m^6^A site in the 3′-UTR of p21, which modulated the stability of p21 mRNA by MeRIP and luciferase reporter assays. METTL3 negatively regulated the expression of p21 by influencing the mRNA stability of p21 and the antiproliferation activity induced by knockdown of METTL3 was rescued by silencing p21, which suggested that p21 was the main target of METTL3 in breast cancer. P21, also known as cyclin-dependent kinase inhibitor 1 (CDKN1A), is a direct target of p53 and mediates G1 growth arrest^47,48^. Metformin was reported to suppress liver cancer cell growth through induction of cell cycle G1/G0 phase arrest by increasing the expression of p21^49^. These studies support our finding that as the downstream target of METTL3 mediated m^6^A modification, p21 was involved in the pathway of the antiproliferation effect of metformin.

In summary, we provided compelling evidences showing that METTL3 markedly promoted breast cancer cell proliferation and high expression of METTL3 tended to be associated with poor clinical outcome in human breast patients. As a well-known candidate for drug repurposing in breast cancer, metformin exhibited antiproliferation activity in breast cancer via the miR-483-3p/METTL3/m^6^A/p21 pathway. Our findings suggest that METTL3 may be considered as a novel potential therapeutic target of metformin for breast cancer.

## Supplementary information

Supplementary information

Figure S1

Figure S2

Figure S3

Figure S4

Figure S5

Table S1

Table S2

Table S3

Table S4

## Data Availability

Please contact the corresponding authors for all data requests.

## References

[1] De, A. & Kuppusamy, G. Metformin in breast cancer: preclinical and clinical evidence. *Curr. Prob. Cancer* 100488, 10.1016/j.currproblcancer.2019.06.003 (2019). 10.1016/j.currproblcancer.2019.06.003 31235186

[2] Coyle C, Cafferty FH, Vale C, Langley RE (2016). Metformin as an adjuvant treatment for cancer: a systematic review and meta-analysis. Ann. Oncol..

[3] Sanchez-Rangel E, Inzucchi SE (2017). Metformin: clinical use in type 2 diabetes. Diabetologia.

[4] Flory J, Lipska K (2019). Metformin in 2019. JAMA.

[5] Evans JM, Donnelly LA, Emslie-Smith AM, Alessi DR, Morris AD (2005). Metformin and reduced risk of cancer in diabetic patients. BMJ.

[6] Thompson AM (2014). Molecular pathways: preclinical models and clinical trials with metformin in breast cancer. Clin. Cancer Res..

[7] Goodwin, P. J. et al. Effect of metformin vs placebo on and metabolic factors in NCIC CTG MA.32. *J. Natl Cancer Inst.* **107**, 10.1093/jnci/djv006 (2015). 10.1093/jnci/djv006 PMC4565534 25740979

[8] Jiralerspong S (2009). Metformin and pathologic complete responses to neoadjuvant chemotherapy in diabetic patients with breast cancer. J. Clin. Oncol..

[9] Dowling RJ, Goodwin PJ, Stambolic V (2011). Understanding the benefit of metformin use in cancer treatment. BMC Med..

[10] Morales DR, Morris AD (2015). Metformin in cancer treatment and prevention. Annu Rev. Med..

[11] Yang Y, Hsu PJ, Chen YS, Yang YG (2018). Dynamic transcriptomic m(6)A decoration: writers, erasers, readers and functions in RNA metabolism. Cell Res..

[12] Zhu W (2019). Detection of N6methyladenosine modification residues (review). Int. J. Mol. Med..

[13] Cai X (2018). HBXIP-elevated methyltransferase METTL3 promotes the progression of breast cancer via inhibiting tumor suppressor let-7g. Cancer Lett..

[14] Miao R (2020). KIAA1429 regulates cell proliferation by targeting c-Jun messenger RNA directly in gastric cancer. J. Cell. Physiol..

[15] Zhang Y (2019). m(6)A modification-mediated CBX8 induction regulates stemness and chemosensitivity of colon cancer via upregulation of LGR5. Mol. Cancer.

[16] Chen M (2018). RNA N6-methyladenosine methyltransferase-like 3 promotes liver cancer progression through YTHDF2-dependent posttranscriptional silencing of SOCS2. Hepatology.

[17] Han J (2019). METTL3 promote tumor proliferation of bladder cancer by accelerating pri-miR221/222 maturation in m6A-dependent manner. Mol. Cancer.

[18] He L (2019). Functions of N6-methyladenosine and its role in cancer. Mol. Cancer.

[19] Huang Y (2019). Small-molecule targeting of oncogenic FTO demethylase in acute myeloid leukemia. Cancer Cell.

[20] Su R (2018). R-2HG exhibits anti-tumor activity by targeting FTO/m(6)A/MYC/CEBPA signaling. Cell.

[21] Yan F (2018). A dynamic N(6)-methyladenosine methylome regulates intrinsic and acquired resistance to tyrosine kinase inhibitors. Cell Res..

[22] Huang Y (2015). Meclofenamic acid selectively inhibits FTO demethylation of m6A over ALKBH5. Nucleic Acids Res..

[23] Visvanathan A (2018). Essential role of METTL3-mediated m(6)A modification in glioma stem-like cells maintenance and radioresistance. Oncogene.

[24] Zhang J (2016). Carbonic anhydrase IV inhibits colon cancer development by inhibiting the Wnt signalling pathway through targeting the WTAP-WT1-TBL1 axis. Gut.

[25] Fukumoto T (2019). N(6)-methylation of adenosine of FZD10 mRNA contributes to PARP inhibitor resistance. Cancer Res..

[26] DeSantis CE (2019). Breast cancer statistics, 2019. CA Cancer J. Clin..

[27] Cheng L (2014). The apoptotic effect of D Rhamnose beta-hederin, a novel oleanane-type triterpenoid saponin on breast cancer cells. PLoS ONE.

[28] Remmele W, Stegner HE (1987). Recommendation for uniform definition of an immunoreactive score (IRS) for immunohistochemical estrogen receptor detection(ER-ICA) in breast cancer tissue. Der. Pathol.

[29] Chen CY, Ezzeddine N, Shyu AB (2008). Messenger RNA half-life measurements in mammalian cells. Methods Enzymol..

[30] Sharma P, Kumar S (2018). Metformin inhibits human breast cancer cell growth by promoting apoptosis via a ROS-independent pathway involving mitochondrial dysfunction: pivotal role of superoxide dismutase (SOD). Cell. Oncol..

[31] Fu Y, Dominissini D, Rechavi G, He C (2014). Gene expression regulation mediated through reversible m(6)A RNA methylation. Nat. Rev. Genet..

[32] Zakikhani M, Dowling R, Fantus IG, Sonenberg N, Pollak M (2006). Metformin is an AMP kinase-dependent growth inhibitor for breast cancer cells. Cancer Res..

[33] Goodwin PJ (2008). Insulin-lowering effects of metformin in women with early breast cancer. Clin. Breast Cancer.

[34] Samuel, S. M., Varghese, E., Kubatka, P., Triggle, C. R. & Busselberg, D. Metformin: the answer to cancer in a flower? Current knowledge and future prospects of metformin as an anti-cancer agent in breast cancer. *Biomolecules* **9**, 10.3390/biom9120846 (2019). 10.3390/biom9120846 PMC6995629 31835318

[35] Wang H, Xu B, Shi J (2020). N6-methyladenosine METTL3 promotes the breast cancer progression via targeting Bcl-2. Gene.

[36] Wu L, Wu D, Ning J, Liu W, Zhang D (2019). Changes of N6-methyladenosine modulators promote breast cancer progression. BMC Cancer.

[37] Wang, Q. et al. METTL3-mediated m(6)A modification of HDGF mRNA promotes gastric cancer progression and has prognostic significance. *Gut*10.1136/gutjnl-2019-319639 (2019). 10.1136/gutjnl-2019-319639 31582403

[38] Li X (2017). The M6A methyltransferase METTL3: acting as a tumor suppressor in renal cell carcinoma. Oncotarget.

[39] Qian JY (2019). KIAA1429 acts as an oncogenic factor in breast cancer by regulating CDK1 in an N6-methyladenosine-independent manner. Oncogene.

[40] Cui Q (2017). m(6)A RNA methylation regulates the self-renewal and tumorigenesis of glioblastoma stem cells. Cell Rep..

[41] Weng H (2018). METTL14 inhibits hematopoietic stem/progenitor differentiation and promotes leukemogenesis via mRNA m(6)A modification. Cell Stem Cell.

[42] Lan Q (2019). The critical role of RNA m(6)A methylation in cancer. Cancer Res..

[43] Blandino G (2012). Metformin elicits anticancer effects through the sequential modulation of DICER and c-MYC. Nat. Commun..

[44] Zhou JY, Xu B, Li L (2015). A new role for an old drug: metformin targets microRNAs in treating diabetes and cancer. Drug Dev. Res..

[45] Huang X, Lyu J (2018). Tumor suppressor function of miR-483-3p on breast cancer via targeting of the cyclin E1 gene. Exp. Therap. Med..

[46] Cui K, Zhang H, Wang GZ (2019). MiR-483 suppresses cell proliferation and promotes cell apoptosis by targeting SOX3 in breast cancer. Eur. Rev. Med. Pharmacol. Sci..

[47] Brugarolas J (1995). Radiation-induced cell cycle arrest compromised by p21 deficiency. Nature.

[48] Deng C, Zhang P, Harper JW, Elledge SJ, Leder P (1995). Mice lacking p21CIP1/WAF1 undergo normal development, but are defective in G1 checkpoint control. Cell.

[49] Cai X (2013). Metformin suppresses hepatocellular carcinoma cell growth through induction of cell cycle G1/G0 phase arrest and p21CIP and p27KIP expression and downregulation of cyclin D1 in vitro and in vivo. Oncol. Rep..

