# Supplementary material for: Metformin exhibits antiproliferation activity in breast cancer via miR-483-3p/METTL3/m6A/p21 pathway
Source: Oncogenesis. 2021 Jan 5;10(1):7. doi: 10.1038/s41389-020-00290-y (PMC7801402; doi:10.1038/s41389-020-00290-y)
Supplement: Supplementary file 1 — Supplementary information [file 41389_2020_290_MOESM1_ESM.docx]

**Supplementary Information**

**Supplementary Results**

**Transcriptome sequencing analysis identified p21 as a downstream target of METTL3.**

To investigate the molecular mechanism and potential targets of METTL3, transcriptome sequencing was performed to assess the expression changes of genes in the stable METTL3 knockdown SUM-1315 cells (Table S3, Figure S3A). Kyoto Encyclopedia of Genes and Genomes (KEGG) analysis revealed that METTL3 could dramatically affect the differently expressed genes (DEGs) enriched in p53 signaling pathway (Figure S3B). Gene Set Enrichment Analysis (GSEA) found that the significantly DEGs induced by METTL3 were mainly concentrated in the p53 signaling pathway (Figure S3C). 22 DEGs involved in the p53 signaling pathway were showed in the heat map (Figure S3D). We further analyzed DEGs from MCF-7 cells treated with metformin (GSE69845) and selected 5 genes as candidate targets of metformin-METTL3 (Figure S3E, Table S4). Next, we detected the 5 genes mRNA expression level in MCF-7 cells. Among the 5 candidates, we found p21 (CDKN1A) was significantly upregulated both in METTL3 knockdown MCF-7 cells and MCF-7 cells treated with metformin. Taken together, p21 was identified as the downstream target of METTL3 (Figure S3F).

**Supplementary files**

**Supplementary file 1: Figure S1.** **METTL3 was upregulated in breast cancer.** (A, B) METTL3 was upregulated in breast cancer cells including SUM1315, MCF-7, BT474 and ZR-75-1, compared with that in the normal breast epithelial cell lines MCF-10A and HBL-100 both in RNA and protein levels. The relative RNA level was calculated by the 2^-ΔΔCt^ method and normalized based on β-actin. The average mRNA level of METTL3 of MCF-10A was set as 1. ^*^*p* < 0.05, ^**^*p* < 0.01, ^***^*p* < 0.001 vs. MCF-10A. (C) Metformin inhibited the growth of MDA-MB-231 cells, however, the cell growth inhibition was weaker than other breast cancer cell lines. (D, E) Western blot and qRT-PCR were used to confirm METTL3 knockdown (shMETTL3-1 and shMETTL3-2) after transfection with lentivirus in SUM-1315, MCF-7 and BT-474 cells. The average mRNA level of METTL3 of shNC group was set as 1. ^**^*p* < 0.01, ^***^*p* < 0.001 vs. shNC group. (F, G) Western blot and qRT-PCR were used to confirm METTL3 overexpression (oeMETTL3) after transfection with lentivirus in SUM-1315, MCF-7 and BT-474 cells. The average mRNA level of METTL3 of oeNC group was set as 1. ^**^*p* < 0.01, ^***^*p* < 0.001 vs. oeNC group. Data represented the mean ± SD.

**Supplementary file 2: Figure S2.** **Overexpression of METTL3 promoted the SUM-1315 and MCF-7 cells proliferation.** (A, B, C) Overexpression of METTL3 significantly increased the growth of in SUM-1315, MCF-7 and BT-474 cells by CCK-8 assay. ^**^*p* < 0.01, ^***^*p* < 0.001 vs. negative control (oeNC). (D, E) Overexpression of METTL3 decreased the colony formation efficiency in SUM-1315 and MCF-7 cells. ^***^*p* < 0.001 vs. negative control (oeNC). (F, G) Cell cycle analyzed by flow cytometry. Histogram showed that METTL3 overexpression decreased the percentage of G1 phase and increased the percentage of G2 and S phases in SUM-1315 and MCF-7 cells. ^**^*p* < 0.01 vs. scramble control group (oeNC). Data represented the mean ± SD.

**Supplementary file 3: Figure S3. Transcriptome sequencing analysis identified p21 as a downstream target of METTL3.** (A) Differentially expressed genes (DEGs) in METTL3 knockdown SUM-1315 cells. (B, C) KEGG and GSEA analysis showed DEGs affected by METTL3 knockdown were mainly concentrated in the p53 signaling pathway. (D) Heat map demonstrated 22 DEGs involved in the p53 signaling pathway. (E) 8 genes were involved in the DEGs from GSE69845 and p53 signaling pathway. (F) The mRNA expression of 5 candidate genes including ATR, BBC3, CASP3, CDKN1A (p21) and THBS1 in the four groups by qRT-PCR. P21 (CDKN1A) was significantly upregulated both in METTL3 knockdown MCF-7 cells and metformin treated MCF-7 cells. The relative RNA level was calculated by the 2^-ΔΔCt^ method and normalized based on β-actin. The average mRNA level of genes mentioned above in the control group and shNC group was set as 1. Control: MCF-7 cells; Metformin: MCF-7 cells treated with metformin; shNC, scramble control MCF-7 cells; shMETTL3-2: METTL3 knockdown MCF-7 cells. Data represented the mean ± SD, ^**^*p* < 0.01, ^***^*p* < 0.001 vs. control group, ^#^*p* < 0.05, ^###^*p* < 0.001 vs. shNC group.

**Supplementary file 4: Figure S4.** **Overexpression of METTL3 downregulated the expression of p21.** (A, B) The mRNA and protein expression of p21 was downregulated after METTL3 overexpression in SUM-1315 and MCF-7 cells. The relative RNA level was calculated by the 2^-ΔΔCt^ method and normalized based on β-actin. The average mRNA level of p21 in oeNC group was set as 1. ^***^*p* < 0.001 vs. oeNC group. (C, D) Overexpression of METTL3 shortened the half-life of p21 transcript in SUM-1315 and MCF-7 cells. (E) Metformin increased the protein expression of METTL3 in SUM-1315, MCF-7 and BT-474 cells in a dose-dependent manner. (F, G) The mRNA level of METTL3 in the tumors from METTL3 overexpression cells significant increased than those from control cells, while the mRNA level of p21 in the tumors from METTL3 overexpression cells significant decreased than those from control cells. (H) MiR-483-3p expression level in breast cancer cells and normal mammary epithelial cells MCF-10A and HBL-100. The relative RNA level was calculated by the 2^-ΔΔCt^ method and normalized based on β-actin. The average mRNA levels of METTL3 and p21 in oeNC group were set as 1. ^*^*p* < 0.05 vs. oeNC group. Data represented the mean ± SD.

**Supplementary file 5: Figure S5. Mode pattern of the pathway miR-483-3p/METTL3/m^6^A/p21 involved in antiproliferation effect of metformin in breast cancer.**

**Supplementary file 6: Table S1.** **Oligonucleotide sequences used in the study.**

**Supplementary file 7: Table S2.** **The association between METTL3 expression and clinicopathologic features in breast cancer.**

**Supplementary file 8: Table S3.** **Differentially expressed genes in METTL3 knockdown SUM-1315 cells.**

**Supplementary file 9: Table S4. Analysis about DEGs of metformin treatment and METTL3 knockdown in breast cancer cells.**
