# Supplementary figures and images for: Metformin exhibits antiproliferation activity in breast cancer via miR-483-3p/METTL3/m6A/p21 pathway
Source: Oncogenesis. 2021 Jan 5;10(1):7. doi: 10.1038/s41389-020-00290-y (PMC7801402; doi:10.1038/s41389-020-00290-y)

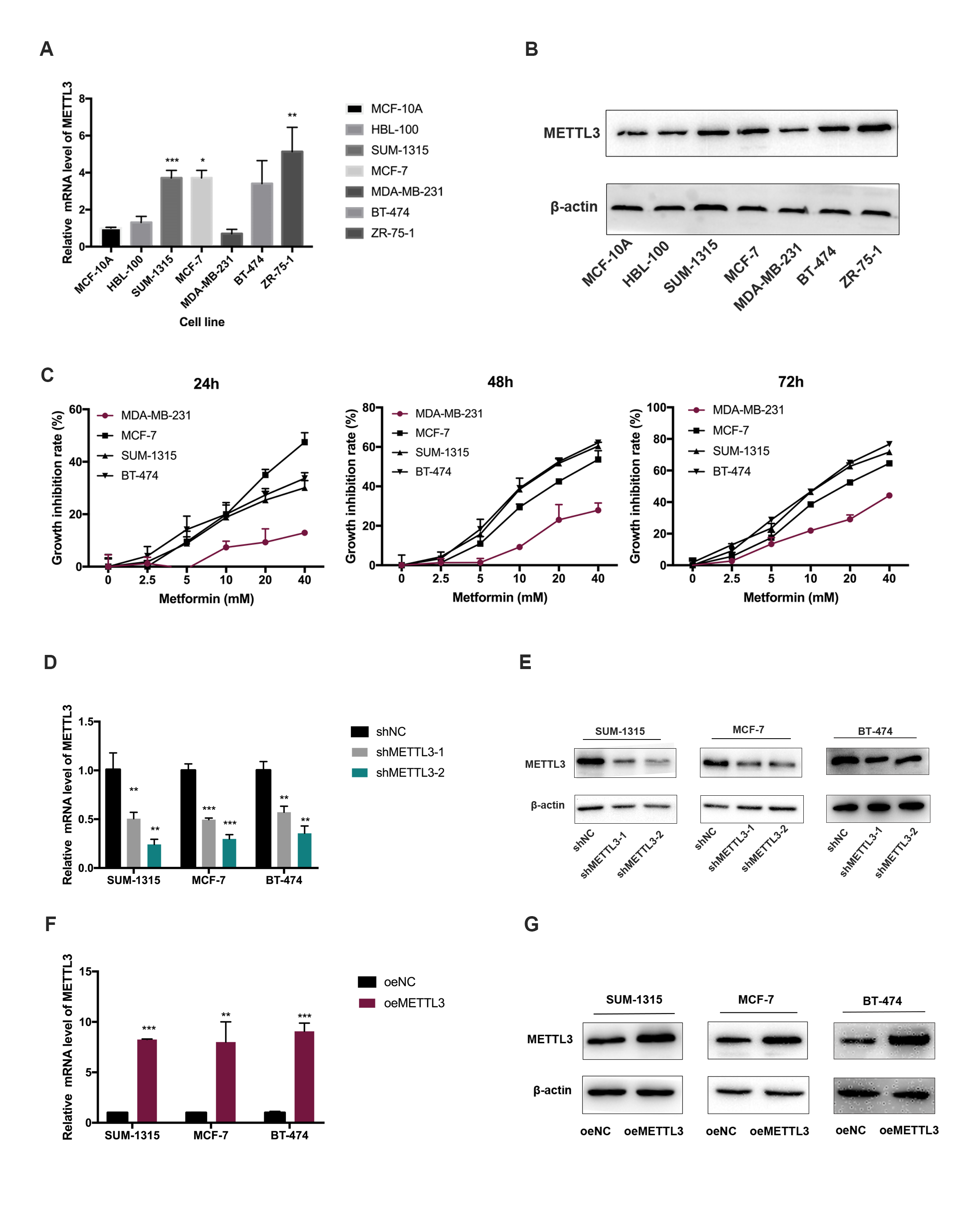

Supplement: Supplementary file 2 — Figure S1 [file 41389_2020_290_MOESM2_ESM.tif]

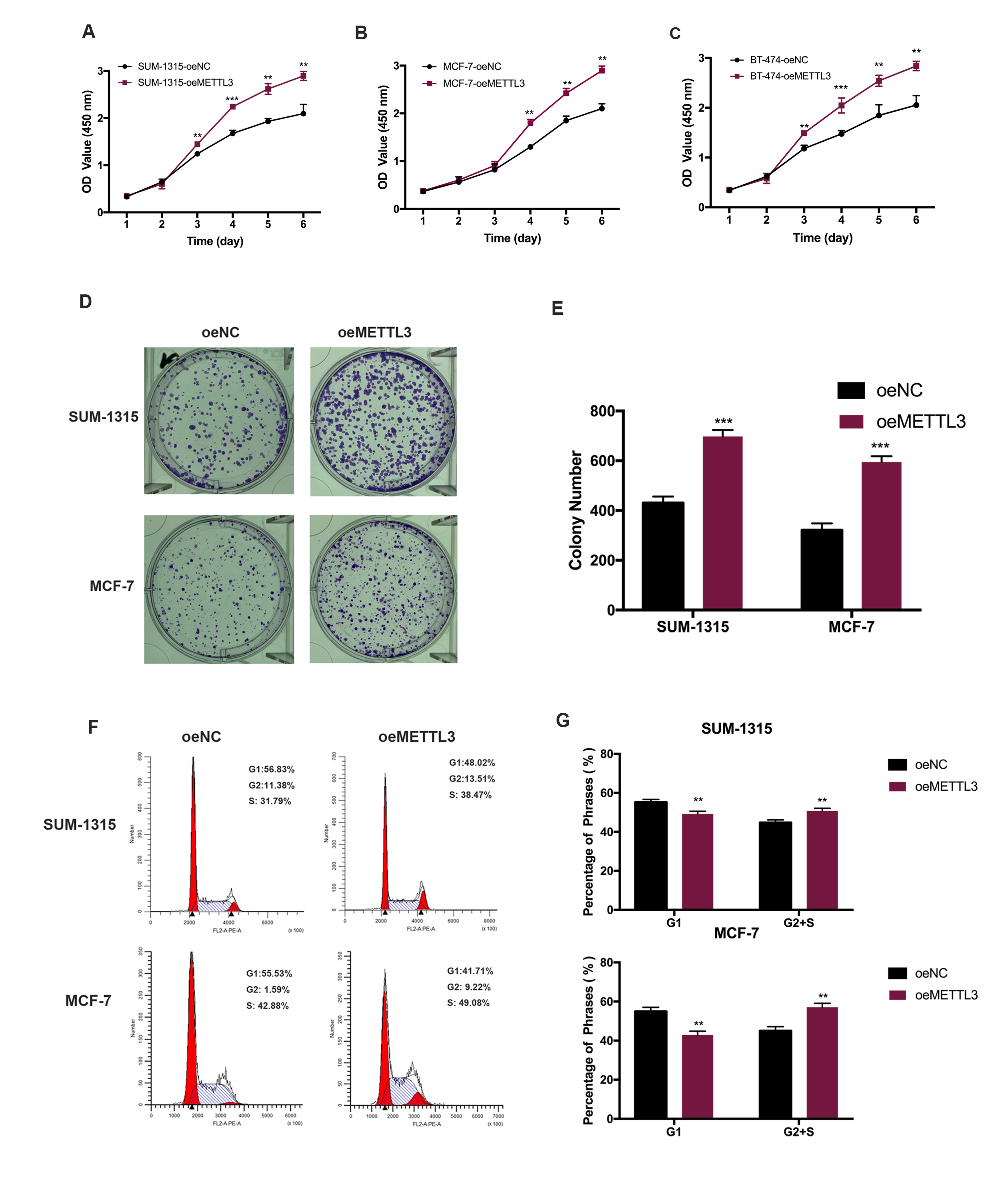

Supplement: Supplementary file 3 — Figure S2 [file 41389_2020_290_MOESM3_ESM.tif]

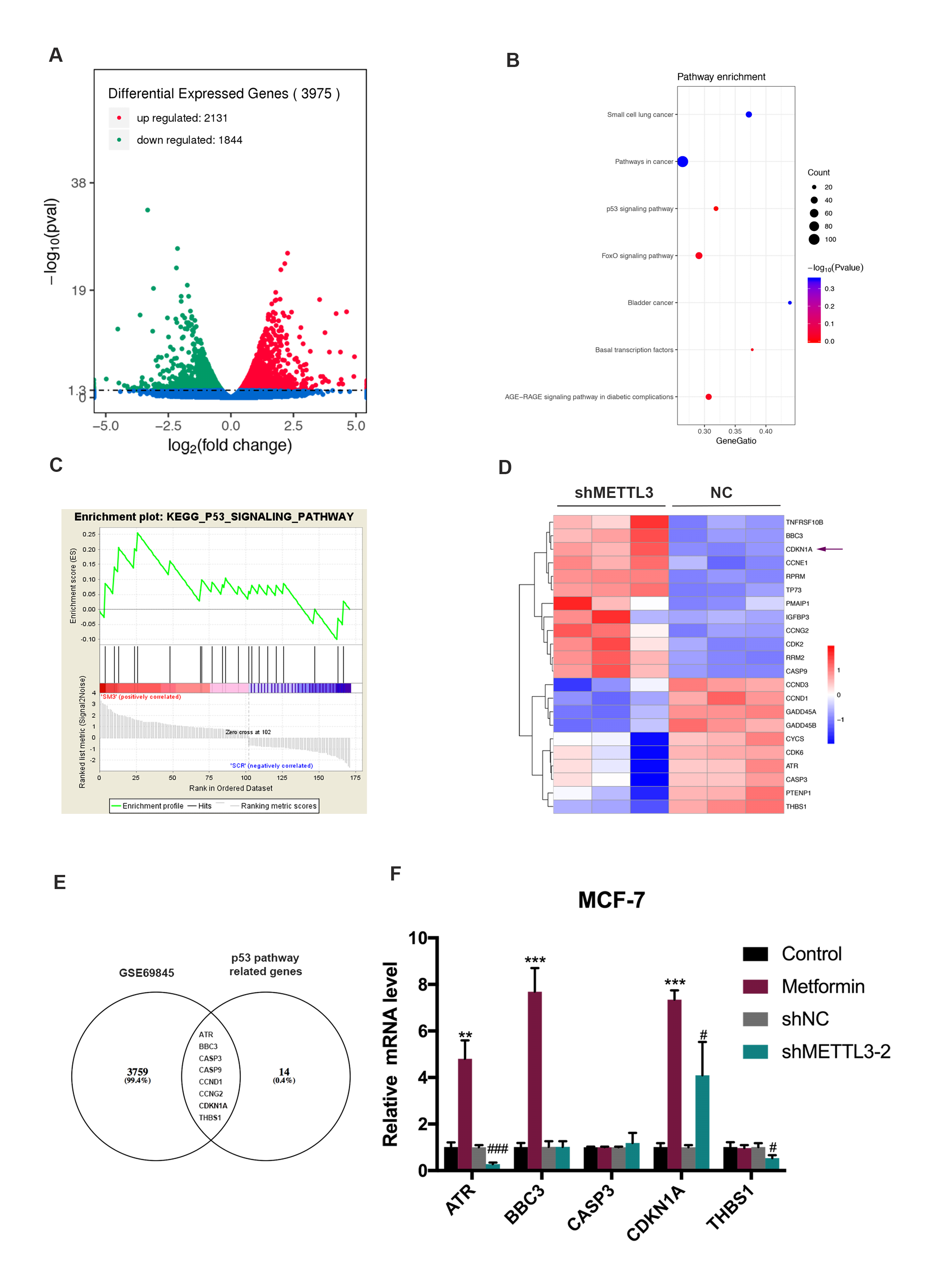

Supplement: Supplementary file 4 — Figure S3 [file 41389_2020_290_MOESM4_ESM.tif]

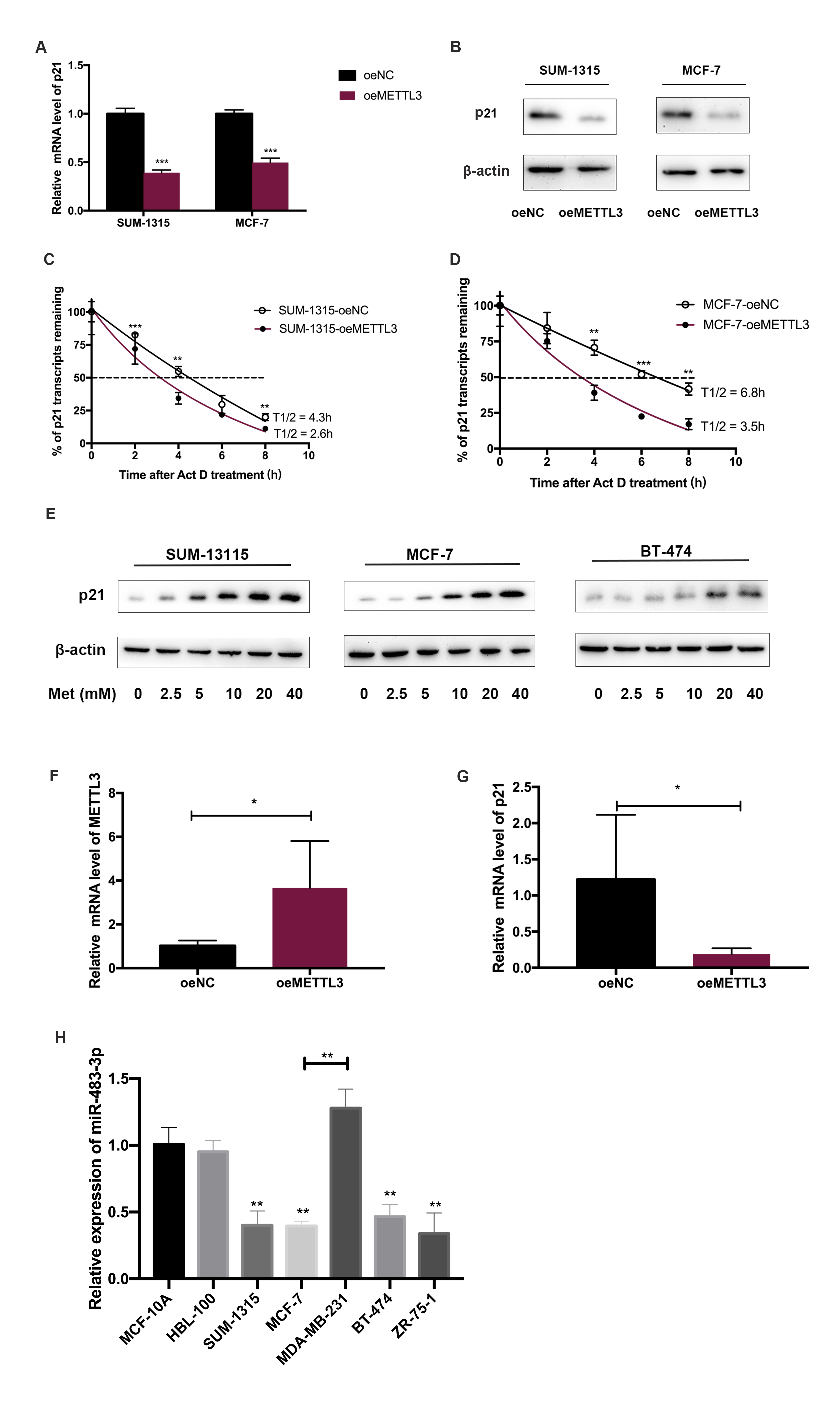

Supplement: Supplementary file 5 — Figure S4 [file 41389_2020_290_MOESM5_ESM.tif]

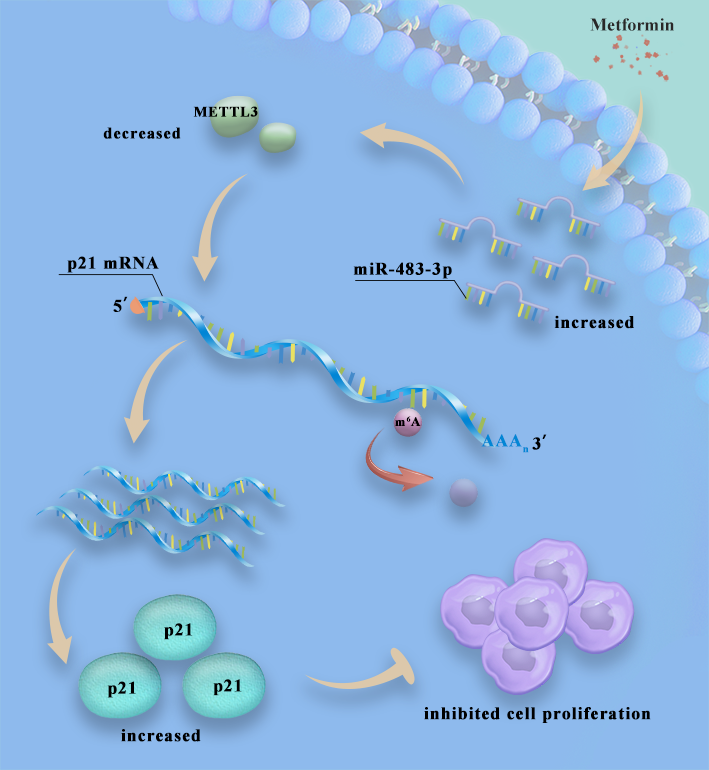

Supplement: Supplementary file 6 — Figure S5 [file 41389_2020_290_MOESM6_ESM.tif]
