# Supplementary material for: Metformin exhibits antiproliferation activity in breast cancer via miR-483-3p/METTL3/m6A/p21 pathway
Source: Oncogenesis. 2021 Jan 5;10(1):7. doi: 10.1038/s41389-020-00290-y (PMC7801402; doi:10.1038/s41389-020-00290-y)
Supplement: Supplementary file 7 — Table S1 [file 41389_2020_290_MOESM7_ESM.docx]

**Table S1** Oligonucleotide sequences used in the study

| Primes and probes |  | |  | | Sequences |
| --- | --- | --- | --- | --- | --- |
| Hsa-miR-483-3p(mimics) | | Forward | | 5′- GAGCUUAUUCAUAAAAGUGCAG -3′ | |
|  |  | Reverse | | 5′- CUGCACUUUUAUGAAUAAGCUC -3′ | |
| NC(mimics) | | Forward | | 5′- UUUGUACUACACAAAAGUACUG -3′ | |
|  |  | Reverse | | 5′- CAGUACUUUUGUGUAGUACAAA -3′ | |
| Hsa-miR-483-3p(inhibitor) | |  | | 5′- AAGACGGGAGGAGAGGAGUGA -3′ | |
| Inhibitor NC | |  | | 5′- CAGUACUUUUGUGUAGUACAAA -3′ | |
| METTL3  METTL14  WTAP  FTO  ALKBH5 | | Forward | | 5′- TGTCCATCTGTCTTGCCATC -3′ | |
|  |  | Reverse  Forward  Reverse  Forward  Reverse  Forward  Reverse  Forward  Reverse | | 5′- GAGAGCTTGGAATGGTCAGC-3′  5′-ATAGCCGCTTGCAGGAGAT -3′  5′-TTTAACACGGCACCAATGC -3′  5′- TTGCCCAACTGAGATCAACA-3′  5′-GCGTAAACTTCCAGGCACTC-3′  5′-GCCTTTCTCACACTGCACAA-3′  5′-CCGTAAAGAGCCTGGTGTTC-3′  5′-TTCAAGCCTATTCGGGTGTC-3′  5′-GGCCGTATGCAGTGAGTGAT-3′ | |
| β-Actin | | Forward | | 5′-ACTGGAACGGTGAAGGTGAC-3′ | |
|  |  | Reverse | | 5′-AGAGAAGTGGGGTGGCTTTT-3′ | |
| p21  ATR  BBC3  CASP3  THBS1 | | Forward | | 5′- TTTCCCTTCAGTACCCTC -3′ | |
|  |  | Reverse  Forward | | 5′- CTTCCAGTCCATTGAGCT -3′  5′- AACTGGACCTGGAGGCAAC-3′ | |
|  |  | Reverse  Forward  Reverse  Forward  Reverse  Forward  Reverse | | 5′- GCCATCATCAGAATGGGAAT-3′  5′- GACGACCTCAACGCACAGTA-3′  5′-ACATGGTGCAGAGAAAGTCC-3′  5′-GAGGCCGACTTCTTGTATGC-3′  5′-AATTCTGTTGCCACCTTTCG-3′  5′-TCCTGGACTCGCTGTAGGTTA-3′  5′-TCACAGAAAGGCCCGAGTAT-3′ | |
