# Supplementary material for: Metformin exhibits antiproliferation activity in breast cancer via miR-483-3p/METTL3/m6A/p21 pathway
Source: Oncogenesis. 2021 Jan 5;10(1):7. doi: 10.1038/s41389-020-00290-y (PMC7801402; doi:10.1038/s41389-020-00290-y)
Supplement: Supplementary file 8 — Table S2 [file 41389_2020_290_MOESM8_ESM.docx]

**Table S2** The association between METTL3 expression and clinicopathologic features in breast cancer (90)

| Variables | METTL3 | | | |
| --- | --- | --- | --- | --- |
|  | Low  expression | High  expression | chi-square  value | *p* value |
| Age (y) |  |  |  |  |
| ≤50 | 14 | 26 | 3.937 | 0.058 |
| >50 | 28 | 22 |  |  |
| Tumor size |  |  |  |  |
| ≤2 cm | 28 | 17 | 8.750 | **0.006** |
| >2cm | 14 | 31 |  |  |
| Not available | |  |  |  |
| Axillary node status | |  |  |  |
| Negative | 24 | 27 | 0.007 | 1 |
| Positive  Not available | 18 | 21 |  |  |
| ER status |  |  |  |  |
| Negative | 16 | 21 | 0.296 | 0.67 |
| Positive | 26 | 27 |  |  |
| Not available |  |  |  |  |
| PR status |  |  |  |  |
| Negative | 16 | 17 | 0.069 | 0.829 |
| Positive | 26 | 31 |  |  |
| Not available |  |  |  |  |
| Her-2 status |  |  |  |  |
| Negative  Positive | 25 | 30 | 0.299 | 0.649 |
|  | 15 | 14 |  |  |
| Not available | 2 | 4 |  |  |
| Ki-67  ≤14%  >14% | 20  22 | 16  32 | 1.905 | 0.199 |
| Grade |  |  |  |  |
| I-II | 15 | 25 | 2.431 | 0.14 |
| II-III | 27 | 23 |  |  |
| TNM stage |  |  |  |  |
| I | 21 | 11 | 7.171 | **0.009** |
| II+III | 21 | 37 |  |  |
| TNBC |  |  |  |  |
| TNBC | 38 | 40 | 0.529 | 0.678 |
| Not TNBC | 2 | 4 |  |  |
| Not available | 2 | 4 |  |  |
| P21  Low expression  High expression | 31  11 | 25  23 | 4.498 | **0.034** |
