# Supplementary material for: Metformin exhibits antiproliferation activity in breast cancer via miR-483-3p/METTL3/m6A/p21 pathway
Source: Oncogenesis. 2021 Jan 5;10(1):7. doi: 10.1038/s41389-020-00290-y (PMC7801402; doi:10.1038/s41389-020-00290-y)
Supplement: Supplementary file 10 — Table S4 [file 41389_2020_290_MOESM10_ESM.docx]

**Table S4 Analysis about DEGs of metformin treatment and METTL3 knockdown in breast cancer cells**

| Genes | Effect | |
| --- | --- | --- |
|  | Metformin  （GSE69845） | METTL3 knockdown  (RNA-seq) |
| **ATR** | decreased | decreased |
| **BBC3** | increased | increased |
| **CASP3** | decreased | decreased |
| CASP9 | decreased | increased |
| CCND1 | increased | decreased |
| CCNG2 | decreased | increased |
| **CDKN1A** | increased | increased |
| **THBS1** | decreased | decreased |
